# The release of tryptase from mast cells promote tumor cell metastasis via exosomes

**DOI:** 10.1186/s12885-019-6203-2

**Published:** 2019-10-29

**Authors:** Hui Xiao, Mudan He, Guogang Xie, Yanan Liu, Yuxia Zhao, Xiong Ye, Xingjing Li, Min Zhang

**Affiliations:** 1 Department of Respiratory and Critical Care Medicine, Shanghai General Hospital, Shanghai Jiaotong University, 85 Wujin Road, Shanghai, 200080 China; 2 Department of Respiratory and Critical Care Medicine, Shanghai General Hospital of Baoshan Branch, Shanghai, China; 3 Department of Clinical Laboratory, Shanghai Children’s Hospital, Shanghai Jiaotong University, Shanghai, China; 4 College of Clinical Medicine, Shanghai University of Medicine & Health Science, Shanghai, China

**Keywords:** Lung cancer, Exosomes, Mast cell, Tryptase, Angiogenesis

## Abstract

**Background:**

Cancer cells release exosomes and can be taken up by mast cells (MCs), but the potential functional effects of MCs on tumor metastasis remain unknown.

**Method:**

Exosomes were isolated from the lung adenocarcinoma cell line A549, and the uptake of PKH26-labeled exosomes by bone marrow MCs was examined via flow cytometry and fluorescence microscopy. Cytokines and tryptase in MC supernatant were analyzed using an ELISA kit, and the presence of tryptase was evaluated by Western blotting. Cell proliferation and migration were determined through CCK-8 and transwell assays. Proteins in the tryptase-JAK-STAT signaling pathway were detected by Western blotting.

**Results:**

In this study, we show that exosomes from A549 cells can be taken up by MCs. Moreover, A549 exosomes contain stem cell factor (SCF) to MCs and subsequently induce the activation of MCs through SCF-KIT signal transduction, which leads to MC degranulation and the release of tryptase. Tryptase accelerates the proliferation and migration of human umbilical vein endothelial cells (HUVECs) through the JAK-STAT signaling pathway.

**Conclusions:**

Our results reveal a mechanism for metastasis in which exosomes can transfer SCF to and activate MCs, which can affect the release of tryptase and the angiogenesis of HUVECs.

## Highlights

Exosomes derived from lung cancer cells possess SCF for binding to mast cells via KIT.

Mast cells release tryptase and are central mediators responsible for the progression of angiogenesis.

Exosomes can promote angiogenesis and tumor metastasis.

## Background

Metastasis is the leading cause of lung cancer-related deaths. Angiogenesis or vascular permeability is a characteristic of the premetastatic niche that enables tumor cell colonization and promotes metastasis. Organs of future metastasis are selectively and actively modified by the primary tumor before metastatic spread [1]. Through complex cross-talk among primary tumor-derived factors and local stromal components, primary tumors create a favorable microenvironment in secondary organs for subsequent metastases [2]. Sowing the ‘seeds’ of metastasis requires tumor-shed exosomes that enable the ‘soil’ at distant metastases promote the capture and growth of circulating tumor cells [1]. Pancreatic ductal adenocarcinoma-derived exosomes initiate premetastatic niche formation in the liver [3]. Moreover, tumor-conditioned lymphatic endothelial cells promote angiogenesis in these organs for breast cancer metastasis [4].

Exosomes are nanosized lipid bilayer membrane vesicles (30–150 nm) that can released by various cells, such as mast cells (MCs) [5], dendritic cells [6], tumor cells [7, 8] and stem cells [9]. Exosomes are well known to transfer their contents, including shuttle functional RNA [10], proteins [11] and lipids [12] between cells. Importantly, the transfer of these molecules can alter the tumor microenvironment [13, 14] and play an important role in intercellular communication within the extracellular environment.

Emerging evidence shows that exosomes derived from tumor cells, including cells from lung cancer [15, 16], colon cancer [17, 18], melanoma [19–21], prostate cancer [22], breast cancer [4, 23] and pancreatic cancer [24] can play an important role in the interplay between immunocytes and tumor cells. Importantly, exosomes derived from lung cancer cells play key roles in tumor loading during metastatic cell seeding [25]. A great deal of evidence points to MCs having key roles in the development of metastases. Mast cell-derived KIT acts as a functional protein that interacts with tumor cells via exosomes and subsequently activates KIT-SCF signal pathway, which accelerates the proliferation in lung cancer cells [11]. However, little is known regarding the immediate fate of incoming lung cancer cell-derived exosomes as they first contact MCs, and even less is known regarding what happens in these exosome-treated MCs. Furthermore, the mechanisms that may allow early-stage lung cancer cell-derived exosomes to complete the pretransfer from the microenvironment to MCs are unknown.

## Methods

### BMMCs

​Bone marrow-derived MCs (BMMCs) were prepared as previously described [26, 27]. BMMCs were cultured in Roswell Park Memorial Institute (RPMI) 1640 medium (Corning, USA) supplemented with 10% heat-inactivated fetal bovine serum (FBS) and 10 ng/ml recombinant interleukin-3 (rIL-3) (PeproTech, USA). Subsequently, the cells were harvested and observed to consist of 98% pure MCs as assessed by toluidine blue staining, CD117 and IgE receptor (FcεRI) expression, confirming that BMMCs can be cultured and release exosomes [26–28].

### Cell culture

The lung adenocarcinoma cell lines A549 and HUVEC cells were obtained from the American Type Culture Collection (ATCC). A549 cells were maintained in Kaighn’s Modification of Ham’s F-12 Medium (F-12 K medium; Gibco, USA), and HUVEC cells were cultured in Dulbecco’s Modified Eagle Medium (DMEM; Gibco, USA) supplemented with 10% exosome-depleted FBS (Viva Cell Biosciences, Qipeng, Shanghai, China) and 100 U/ml penicillin and 100 μg/ml streptomycin. The cells were maintained in a humidified incubator at 37 °C with 5% CO_2_.

### Isolation of exosomes

The A549 cell culture media were collected 3 days after the start of the incubation. The medium was harvested and centrifuged at 1500 rpm for 10 min to remove the cells. Subsequently, the medium was centrifuged at 14,500 RCF for 20 min at 4 °C and then filtered through a 0.2 μm filter (Merck Millipore, Cork, Ireland) to remove cell debris and larger vesicles. Exosomes were sedimentation by ultracentrifugation at 120,000×g for 70 min, and the exosome pellets were resuspended in 150 μl phosphate buffered saline (PBS) and frozen at − 80 °C.

### Transmission electron microscopy analysis

Transmission electron microscopy analyses were performed as previously described [29]. Exosomes from A549 cells were loaded onto carbon-coated 200-mesh, thin-bar copper grids and post-fixed in 2.5% glutaraldehyde, washed, contrasted in 2% uranylacetate, embedded in a mixture of uranyl acetate (0.4%), and examined in a LEO 912AB Omega electron microscope (Carl Zeiss NTS, Oberkochen, Germany).

### Degranulation assay of β-hexosaminidase release rate

BMMCs (5 × 10^5^ cells/ml, 0.5 ml) were incubated in a 24-well plate for routine culturing, with three parallel wells used for each group. The cells were centrifuged and resuspended three times in 500 μl Tyrode’s solution. Different concentrations (10 or 50 ng/ml) of human stem cell factor (SCF; PeproTech, USA), 20 μg of exosomes derived from A549 cells or the same amount of PBS were added to the cells, which were subsequently incubated at 37 °C for 24 h. The exosome group included four subgroups that were incubated for 4, 8, 12 or 24 h. The reactions were terminated after the cells were incubated for 10 min in an ice bath. The supernatant of each well was transferred into a 96-well plate, and the absorbance of each well was measured at 405 nm after adding 50 μl of the substrate (p-nitrophenyl-N-acetyl-beta-D-glucosaminide; Aladdin, China), incubating at 37 °C for 60 min and then adding 150 μl of the stop buffer (200 mmol/L glycine, pH 10.4). After discarding the supernatant, 200 μl Triton X-100 (0.5%) was added to each well (30 min), after which the lysate was centrifuged at 10,000×g for 30 min. Subsequently, the absorbance of the lysate supernatant was determined for each sample. The release of β-hexosaminidase (%) was reported as the supernatant absorbance divided by that of the supernatant of the cell lysate.

### Uptake of A549 exosomes by BMMCs

To monitor exosome uptake kinetics, exosomes derived from A549 cells were labeled with the red fluorescent dye PKH26 (Sigma-Aldrich). In brief, isolated exosomes from A549 cells labeled PKH26 dye(20μg) were washed three times using 100 kDa Vivaspin filters (Millipore, USA) to eliminate excess dye and were then added to 2.5 × 10^5^ cells/ml BMMCs cultured on the confocal plate. PBS was mixed with PKH26 and added to the cells as a control for nonspecific labeling. BMMCs were harvested at different time points (1, 2, 4, 8, 12 and 24 h) and analyzed by flow cytometry. For the flow cytometry analysis, BMMCs were washed twice with PBS and treated with a 0.25% trypsin to detach the cells. Subsequently, the cells were washed three with 1% BSA-PBS acquired in Beckman Coulter FC500 instruments and analyzed with FlowJo software. For fluorescence microscopy (DMi8; Leica, GER), BMMCs were washed twice with PBS, and fixed with a 4% formaldehyde solution for 15 min and then washed again twice with PBS. The cells were supplemented with a DAPI staining solution (Beyotime, China) to label cell nuclei.

### Determination of cytokine levels

BMMCs (5 × 10^5^ cells/ml, 2 ml) were incubated in the 6-well cell culture plate with media overnight. Subsequently, medium, SCF (100 ng) and A549 cell-derived exosomes (40 μg) were added to BMMCs. After incubating for 24 h, the cell supernatants were collected in ultrafiltration tubes (50, 10, or 3 kDa; Millipore, USA) according to the formula weight of the cytokines. The cell supernatants were collected for ELISA after centrifuging at 2000×g for 6 min. The levels of tryptase, interleukin-6 (IL-6), matrix metallopeptidase-9 (MMP-9) and tumor necrosis factor alpha (TNF-α) were determined using an ELISA kit (RND, USA) according to the manufacturer’s instructions.

### Western blot analysis

The supernatant of BMMCs (5 × 10^5^ cells/ml, 2 ml) was collected after incubating overnight and was then incubated with media, SCF (100 ng) and A549 cell-derived exosomes (40 μg) as described for the ELISA test. The protein in the cell supernatants was extracted using methanol and chloroform. After measuring the protein concentration, 20 μg of protein was subjected to SDS-PAGE and transferred to PVDF membranes (GE Healthcare, Piscataway, NJ, USA). PVDF membranes were blocked in 5% bovine serum albumin in Tris-buffered saline with Tween 20 (BSA-TBST) for 2 h. The membranes were then incubated overnight at 4 °C with the primary rabbit anti-human antibodies diluted in 5% BSA-TBST: anti-CD81, anti-SCF and anti-calnexin (Santa Cruz Biotechnology), anti-KIT (Abcam, USA), anti-tryptase (Proteintech, China), anti-JAK, anti-p-JAK, anti-STAT and anti-p-STAT(Cell Signaling Technology Inc). The membranes were washed 3 times for 5 min each before being incubated with the secondary antibody for 2 h. The secondary antibody was goat anti-rabbit IgG (horseradish peroxidase (HRP)-conjugated, Proteintech, China) diluted in 5% BSA-TBST. The membrane was washed 3 times for 5 min each before being analyzed using the ClarityTM ECL Western Blotting Detection System (Bio-Rad Laboratories). The relative intensity for p-JAK and p-STAT was calculated as follows: (phosphorylated protein/GAPDH)/(total protein/GAPDH).

### Detection of cell proliferation

Human umbilical vein endothelial cells (HUVECs; 5 × 10^4^ cells/well) in monoculture were stimulated with tryptase (50 ng/ml) and different supernatants (10 μl) from BMMCs incubated with SCF- and exosomes derived from A549 cells for 24, 48 and 72 h. Cell proliferation was detected using a Cell Counting Kit-8 (CCK-8; Dojindo, Shanghai, China) according to the manufacturer’s protocol. The absorbance was a measured at 450 nm using a spectrophotometer (Spectrum Max; Molecular Devices, Pulang, Beijing, China). The results were normalized as a percent to the control.

### Wound healing assay

HUVECs were cultured in Dulbecco’s modified Eagle’s medium (DMEM) in six-well plates (1 × 10^5^ cells/ml), with 500 μl of cell suspension and 2 ml of FBS-depleted DMEM exosome-complete medium were added to each well. When the cell abundance reached 90%, cells were starved for 12 h with serum-free DMEM. Subsequently, a wound was made in each well with a 200-μl plastic pipette tip. After being washed three times with PBS, the cells were cultured with serum-free DMEM. In addition, a well-balanced transwell chamber (Millipore Corporation, USA; pore size 0.4 um) was placed in the six-well plates. Tryptase (50 ng/ml) and the cell supernatant of SCF- and exosome-stimulated cells were inoculated into the upper chamber of each plate. Cell growth was monitored at 0, 12, 24, 48 and 72 h. The wound width was then observed in each well at each time point and measured using a Leica LAS V3.7 imaging system.

### Statistical methods

The statistical analyses and graphs were generated using GraphPad (GraphPad Software Prism 6, La Jolla, USA). The statistical analyses of western blot were performed using Students t-test. Student’s t-test was used to analyze the differences between two groups, and Kruskal Wallis tests were used to compare among three or more groups. All tests of statistical significance were 2-sided with a significance level set at < 0.05.

## Results

### Identification of lung cancer cell exosomes and BMMCs

**Fig. 1:**
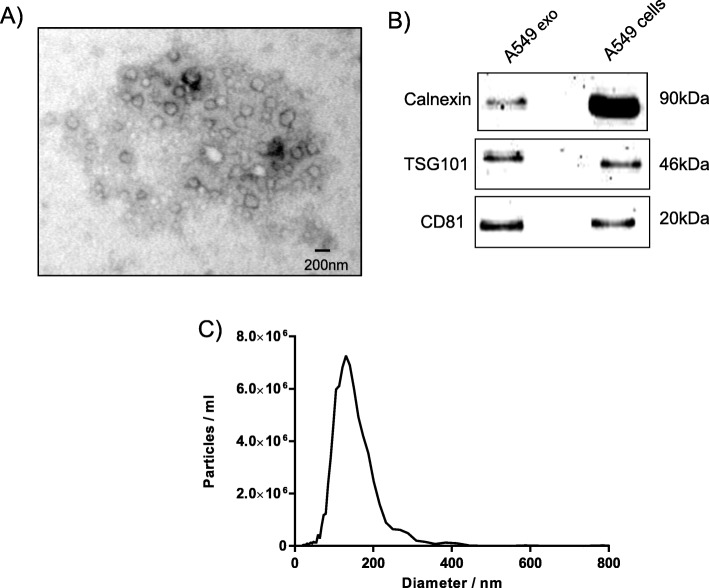
Identification and characterization of A549 cell-derived exosomes. Exosomes were isolated using differential centrifugation. **a** The electron micrographs of the exosomes revealed rounded structures with a size of approximately 30–150 nm. The scale bar represents 200 nm. **b** Western blot analysis of exosomes derived from the supernatant of A549 cells shows the presence of the common exosomes proteins CD81, calnexin and TSG101. Cells were used as a control. **c** The sizes of exosomes derived from the supernatant of A549 cells were analyzed using nanoparticle tracking analysis

Vesicular round structures were visualized by electron microscopy after isolating exosomes from the supernatant of cultured A549 cells using ultracentrifugation (Fig. 1a). The exosome traditional markers were positive for CD81, TSG101 and calnexin (Fig. 1b). The characterization of exosomes by nanoparticle tracking analysis showed an average particle size of approximately 130 nm (Fig. 1c). These exosomes were therefore considered appropriate for use in this study.

**Fig. 2:**
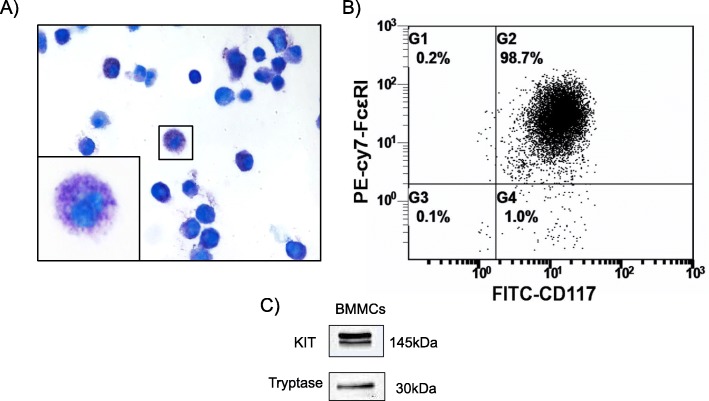
Characteristics of bone marrow mast cells. **a** Bone marrow mast cells (BMMCs) showed abundant purple granules following toluidine blue staining (400×). **b** BMMCs were positive for CD117 and IgE receptor (FcεRI) by flow cytometry analysis. **c** The Western blotting analysis indicated that BMMCs expressed KIT and tryptase

BMMCs were generated in the presence of IL-3 after being cultured for 4 weeks. Figure 2a shows the morphology of BMMCs containing an abundance of purple granules after toluidine blue staining. Flow cytometry analysis was also performed to identify MCs based on the expression of CD117 and FcεR1, the results of which suggested that over 98.7% of the cells were MCs (Fig. 2b). BMMCs were positive for the protein markers KIT and tryptase (Fig. 2c), indicating that the cells could be utilized for subsequent experiments.

### Effect of lung cancer cell exosomes on MCs

**Fig. 3:**
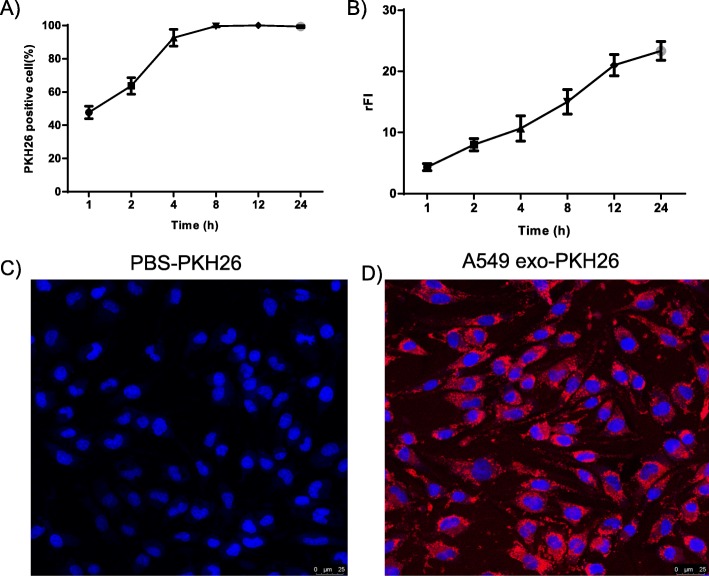
Uptake of A549 cell-derived exosomes by mast cells. **a** and **b** The percent positive cells and relative fluorescence intensity (rFI) data for different time points were determined with flow cytometry for 1–24 h and are shown as the means ± SEM (*n* = 3). **c** and **d**) Uptake of PKH26-PBS control and PKH26-labeled exosomes by fluorescence microscopy imaging. Nuclei were stained with DAPI (blue)

To examine whether exosomes from lung cancer cells can be taken up by MC, exosomes from A549 cells were labeled with PKH26 dye and added to BMMC cultures. Flow cytometry analysis showed an increase in the fluorescence intensity of BMMC after addition of lung cancer cell-derived exosomes, indicating cellular uptake (Fig. 3a). Eight hours after the addition of the stained exosomes, the fluorescence of BMMC was markedly increased with time (Fig. 3b), indicating that BMMC began exosomal uptake. Uptake of fluorescent exosomes by BMMC was also observed by fluorescence microscopy (Fig. 3c and d).

**Fig. 4:**
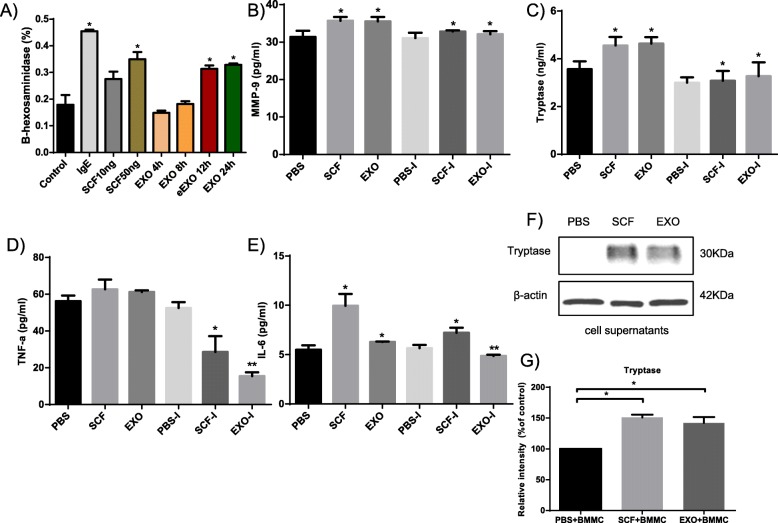
A549 cell-derived exosomes induce mast cell activation, degranulation and tryptase release. **a** Mast cells were activated with A549 cell-derived exosomes carrying SCF, which led to an increased rate of hexosaminidase release. The release of cytokines into the supernatant of mast cells in the presence of A549 cell-derived exosomes at 24 h was detected by ELISA. MMP-9 (**b**), tryptase (**c**), TNF-α (**d**) and IL-6 (**e**) were significantly increased in the experimental group, which was significantly different from the control and inhibitor groups. **f** The supernatants of mast cells stimulated with SCF or A549 cell-derived exosomes were analyzed for tryptase and β-actin via Western blotting. **g** Relative intensity was calculated for tryptase. All of the above data are representative of three independent experiments (n = 3). *P*-values, * < 0.05, ** < 0.01

To determine whether exosomes from lung cancer cells influence MCs, the presence of specific proteins and cytokines was assessed in the supernatants of MCs in the presence of A549 cell-derived exosomes. First, A549 cell-derived exosomes were added to BMMCs in culture at different time points (4, 8, 12 and 24 h), and the release of β-hexosaminidase was used to evaluate the extent of degranulation (Fig. 4a). The percentage of BMMCs degranulation was significantly increased (by 200%) in response to the treatment with A549 cell-derived exosomes (20 μg) for 24 h, a result that is consistent with the optimal incubation time described above. SCF (10 ng and 50 ng) and IgE were used as positive controls. The production of MMP-9, tryptase, IL-6 and TNF-α in cell supernatants was significantly increased by the treatment of BMMCs with exosomes compared to the PBS control and inhibitor, as quantified using an ELISA kit (*P* < 0.05) (Fig. 4b-e). Second, the MC supernatants were positive for the protein tryptase in the presence of A549 cell-derived exosomes or SCF (Fig. 4f) compared with the negative control (Fig. 4g).

### Detection of tryptase in MC supernatants and activation of the JAK-STAT signaling pathway in HUVECs

**Fig. 5:**
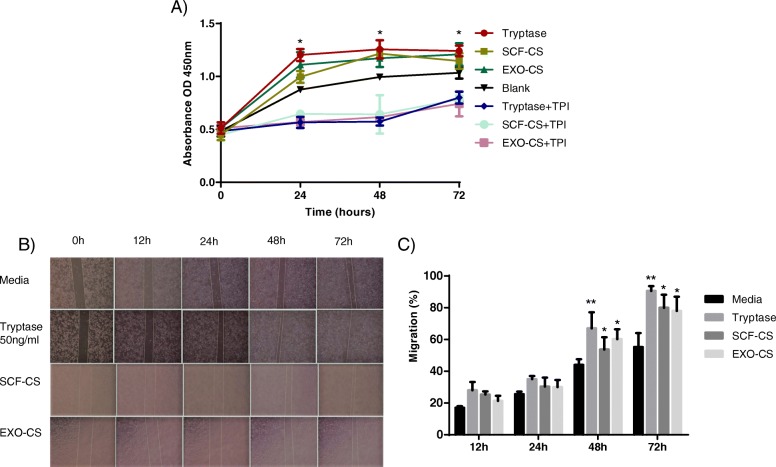
Mast cell-released tryptase induces HUVECs proliferation and migration. **a** CCK-8 cell proliferation assays were performed to detect HUVECs proliferation after co-culturing for 24–72 h with mast cell supernatant or control reagents (SCF and blank). *P < 0.05. **b** and **c** HUVECs were added to the lower transwell chamber, while the upper chamber contained 20 μl of supernatant from mast cells. Tryptase- and SCF-stimulated mast cell supernatants were used as controls. After 12–72 h, the number of cells that migrated was analyzed by taking photos and counting the area per visual field. *P*-values, * < 0.05, ** < 0.01

To determine whether supernatants of BMMCs stimulated by SCF or A549 cell-derived exosomes affected HUVECs, the cell supernatants were added to cultured HUVECs for 24, 48, and 72 h in the presence of CCK-8 to evaluate their effect on cell proliferation. The proliferation of HUVECs was significantly enhanced on the presence of tryptase, cell supernatants stimulated by A549 cell-derived exosomes or SCF compared to that of cells treated with the control medium and the inhibitor, as quantified using an ELISA kit (Fig. 5a).

To examine the effects of the supernatants of BMMCs stimulated by SCF or A549 cell-derived exosomes on HUVECs, HUVECs were seeded onto the membrane of a upper transwell chamber and with tryptase, SCF-cell supernatant (CS) and exosome (EXO)-CS present in the lower chamber to evaluate the capacity of tryptase to induce migration. Significantly more cells migrated into the lower chamber in the presence of tryptase than was observed in the control at 48 and 72 h (Fig. 5b and c).

**Fig. 6:**
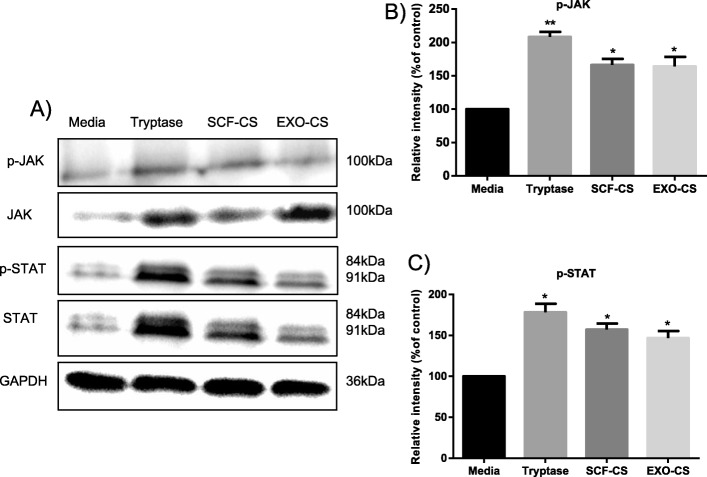
Mast cell supernatant activates the JAK-STAT signaling pathway in HUVECs. **a** HUVECs treated with supernatants from mast cells stimulated with SCF or A549 cell-derived exosomes were analyzed via Western blotting and were compared with HUVECs treated with medium and tryptase. Phosphorylated (p)-JAK and p-STAT and total JAK and STAT were measured by Western blotting and protein loading was normalized, with the samples also probed for GAPDH. **b** and **c** Relative intensity was calculated for p-JAK and p-STAT. All of the above data are representative of three independent experiments (n = 3). *P < 0.05 and **P < 0.01

To propose a molecular model for the observed proliferation and migration of HUVECs, we attempted to identify the pathways activated by the addition of tryptase. As shown in Fig. 6a, we observed increased phosphorylation of JAK and STAT when tryptase, SCF-CS, and EXO-CS were added compared to that with media alone. Furthermore, the total amounts of JAK and STAT were unchanged according to the analysis of the Western blot. Thus, tryptase, SCF-CS and EXO-CS may activate the proliferation of HUVECs through the JAK-STAT pathway.

## Discussion

Metastasis is one of the basic characteristics of tumors. Tumor tissues contain many immune cells, including MCs. It has been suggested that exosomes can shuttle RNA, microRNA and proteins between cells, which is a process that is likely to be highly active in cell-to-cell signaling in tumors. However, little is known regarding how MCs may act to specifically promote cancer metastasis. In this study, we observed that exosomes derived from the lung adenocarcinoma cell line A549 contain the SCF receptor, which can be transferred to MCs via exosomes. Specifically, we showed that lung cancer cells release exosomes with typical exosome markers, such as TSG101, CD81 and calnexin. The lung cancer cell-released exosomes were rapidly taken up by BMMCs, a process that peaks after approximately 12 h. Importantly, BMMCs were observed to contain the natural SCF receptor KIT, which induced the activation and degranulation of BMMCs. The release of tryptase promotes the proliferation and migration of HUVECs to form the premetastatic microenvironment for lung cancer. Furthermore, tryptase derived from MCs enhanced the JAK-STAT signaling pathway activity. Overall, these data suggest that exosomes from lung cancer cells enhance the proliferation of HUVECs by activating MCs and the SCF-KIT-induced release of tryptase, potentially by enhancing JAK-STAT signaling in HUVECs.

In this study, we confirmed the ability of lung cancer cells to release exosomes into their microenvironment and their uptake by BMMCs. The electron micrographs and nanoparticle tracking analysis of exosomes revealed the presence of rounded structures with a size of approximately 30–150 nm, similar to previously described reports [30, 31]. Exosomes derived from lung cancer cells had typical exosome markers, such as CD81, TSG101 and calnexin, which fits with the previously reported protein content for exosomes. The observed uptake was rapid, as more than 45% of cells had already taken up some exosomes after one hour, and almost all cells were positive for exosomes after eight hours. However, the uptake of exosomes continued over time, as measured by the relative fluorescence intensity, and peaked at 12–24 h after the addition of exosomes. This time course of uptake was slightly slower than what we have previously reported [32].

Although endogenously produced exosomes may have a role in tumors, the associated mechanism is not fully understood. As expected, A549 cell-derived exosomes activated the release of proinflammatory mediators, such as β-hexosaminidase, tryptase, MMP-9, TNF-α and IL-6 from activated MCs. To exclude the possibility of the influence of other cytokines and proteins from the cell supernatants, we assayed the effect of tryptase from the cell supernatants in the current study. Protease activated receptor 2 (PAR2) expressed by HUVECs can be activated by serine proteases, such as the MC mediator tryptase. In the current study, we showed that active BMMC-derived tryptase enhanced the proliferation and migration capacity of HUVECs in a coculture system. Based on previously published data on the mitogenic properties of tryptase, we speculated that the changes in HUVEC migration could be due to an increased cell proliferation rate. Accordingly, the binding of tryptase to PAR2 could upregulate the phosphorylation of JAK and STAT, which is regarded as a partial signaling cascade in activated HUVECs.

The shortcomings of this study were activated MCs release many molecules, such as cytokines and proteins that play many key roles in angiogenesis. This study focused only on the effect of tryptase on HUVECs.

Our current series of studies are mainly focused on the SCF-containing exosomes from a lung cancer cell line (A549) and showed that these exosomes can be taken up by MCs. This uptake leads to the activation of MCs, which release tryptase to enhance the proliferation and migration of HUVECs by activating the JAK-STAT signaling pathway. Future work can elucidate the molecular mechanisms leading to the release of exosomes from cancer cells. Furthermore, it is essential to understand the formation of the tumor metastasis microenvironment and the targeting of metastatic organs from different tumor cell-derived exosomes.

## Conclusions

Our results reveal a mechanism for metastasis in which exosomes can transfer SCF to and activate MCs, which can affect the release of tryptase and the angiogenesis of HUVECs activating the JAK-STAT signaling pathway.

## Data Availability

The datasets obtained and/or analyzed during the current study are available from the corresponding author upon reasonable request.

## Abbreviations

BMMCs: Bone marrow mast cells
CS: Cell supernatant
FBS: Fetal bovine serum
GAPDH: Glyceraldehyde 3-phosphate dehydrogenase
HUVECs: Human umbilical vein endothelial cells
SCF: Stem cell factor
